# p53 immunostaining pattern is a useful surrogate marker for *TP53* gene mutations

**DOI:** 10.1186/s13000-022-01273-w

**Published:** 2022-12-05

**Authors:** You-Na Sung, Deokhoon Kim, Jihun Kim

**Affiliations:** Department of Pathology, Asan Medical Center, University of Ulsan College of Medicine, 88, Olympic-Ro 43-Gil, Songpa-Gu, Seoul, 05505 Korea

**Keywords:** TP53 gene, Immunohistochemistry, Mutation, p53 protein, Association

## Abstract

**Background:**

*TP53* is the most frequently mutated gene in the human cancer, and the awareness of its mutational status is useful in the diagnosis and treatment of cancer patients. In the present study, we investigated the association between *TP53* gene mutations and p53 immunohistochemical staining (IHC) patterns and non-genetic effect of MDM2 as a negative regulator of p53.

**Methods:**

A total of 135 solid cancer cases with next generation sequencing data were subjected to p53 IHC and classified as overexpression, null type or usual pattern.

**Results:**

*TP53* mutation was observed in 104 out of 135 cases (77.0%). When the *TP53* mutations were annotated into DISRUPTED (truncations, frameshifts, splice site mutations, and deep deletions) and IF-DBD (in-frame mutations in the DNA binding domain), the null type p53 IHC pattern was associated with DISRUPTED mutations (sensitivity 86.2%, specificity 97.2%) while the overexpression pattern was associated with IF-DBD mutations (sensitivity 100%, specificity 81.7%). The specificity of p53 IHC usual pattern predicting wild type *TP53* was also as high as 100%. Regardless of *MDM2* amplification, p53 IHC pattern showed a perfect association with *TP53* mutation pattern.

**Conclusions:**

p53 IHC pattern (overexpression, null type, usual) reasonably predicted *TP53* mutational status (DISRUPTED, IF-DBD), and *MDM2* amplification status did not have any impact on the p53 IHC pattern.

**Supplementary Information:**

The online version contains supplementary material available at 10.1186/s13000-022-01273-w.

## Background

TP53, functioning as a cellular stress sensor for physiologic or oncogenic activation, is the most frequently mutated gene in human cancer [1–4]. When there is oncogenic activation, DNA replication stress disrupts physiologic function of p53, resulting in cell death evasion and thus, *TP53* mutation is selected during tumor progression [1, 5–7]. Because the p53 is so essential for tumorigenesis and tumor progression, there have been several attempts to elicit tumor cell death by reactivation of mutated *TP53* [8–11]. For instance, a conceptual basis for alleviating the effect of inactivating *TP53* mutation through artificial second-site mutation has been proposed [12, 13]. Furthermore, pathogenic *TP53* mutations are known to be associated with poor survival or chemo-resistance in various tumor types [12, 14–21]. Therefore, it is very important to check the *TP53* mutational status to predict the patient’s prognosis and treatment responsiveness.

Sanger sequencing is known as a traditional gold standard method to confirm the mutational status of *TP53*, but it’s labor intensive and time consuming especially because the *TP53* gene is quite big. Because of these practical problems, it’s difficult to use Sanger sequencing in actual practice, and instead, immunohistochemical staining (IHC) is the most widely used way to infer *TP53* mutational status. Several attempts have been made to infer *TP53* mutation type through p53 IHC pattern, but most previous studies were limited to one specific organ, and the methods of interpreting the p53 IHC results varied, such as pattern-based or positive cell proportions [22–30].

To better understand the association between *TP53* gene mutations and p53 IHC patterns, we used a set of tumor samples having *TP53* gene mutation data across entire *TP53* exons obtained by a clinical grade next-generation sequencing (NGS) test. Through annotation of *TP53* mutations according to the predicted effects on p53 protein function and classification of p53 IHC patterns according to the presence of overexpression or complete loss of expression, we investigated the association between the *TP53* mutation status and p53 IHC pattern. When the p53 IHC pattern and expected *TP53* mutation status were discordant, we analyzed the reasons for the discrepancies. Secondly, we investigated p53 IHC pattern in *MDM2*-amplified cases to determine how p53 IHC appears through the non-genetic effect of MDM2, a negative regulator of p53.

## Methods

### Case selection

After the approval (protocol number, 2018–0976) from the institutional review board, we retrospectively collected 135 adult solid cancer samples that have clinical next-generation sequencing (NGS) data from the records of the Department of Pathology at the Asan Medical Center, University of Ulsan College of Medicine. Among them, 26 cases were obtained after neoadjuvant treatment and 12 cases showed unequivocal *MDM2* amplification.

### Mutational analysis

The clinical NGS cancer panel test was performed as described previously [31]. Briefly, the targeted NGS used the MiSeq platform (Illumina, San Diego, CA) and the gene panel, OncoPanel AMC version 3, was designed in house through SureDesign (Agilent Technologies, Santa Clara, CA) using GRCh37 reference version. This 1.2 Mbp-sized panel included 33,524 probes targeting a total of 382 genes, including entire exons of 199 genes, 184 hot spots, and partial introns for eight genes often rearranged in cancer. Of course, the entire *TP53* and *MDM2* exonic regions were included.

We classified the *TP53* mutations according to the type of nucleotide changes: single nucleotide variation (SNV), in-frame insertion or deletion (In-frame indel), frameshift insertion of deletion (frameshift indel), premature stop codon (truncation), splice site mutation (splice), and copy-number (CN) loss. We classified the *TP53* mutations into two major categories, in-frame alterations across the DNA binding domain (IF-DBD) and alterations involving significant disruption of protein coding sequences (DISRUPTED). The IF-DBD mutations included SNVs and In-frame indel across DNA binding domain. The DISRUPTED mutations included frameshift indel, truncation, splice, and CN loss. The *MDM2* amplifications were detected through our bioinformatics pipeline using CNVkit v0.9.6 [32]. Copy numbers of tumors were called against a panel of normal. Copy number segments were inferred using circular binary segmentation(CBS) method. Absolute copy number of given segments was estimated using ‘call’ module of CNVkit and pathologist’s tumor cellularity. We used pathologist’s tumor purity estimates to infer copy-number more accurately. This involved solving an equation: (measured copy number) = (actual copy number) * (tumor purity) + 2 * (1-tumor purity). To investigate the biologic effect of MDM2 overexpression on TP53 status, we included tumor samples with high level *MDM2* amplifications (estimated *MDM2* copy number > 30). All cases were manually reviewed through visualization of bam files with the integrated genomic browser (IGV) and double check of tumor cell purity through review of microscopic slides and variant allelic fractions of the detected clonal mutations.

### Immunohistochemistry

Representative areas from formalin-fixed, paraffin-embedded tissues were subjected to IHC, which was performed on 4-μm-tick sections using a Ventana auto-stainer and an ultra-View DAB Detection Kit (Ventana, Tucson, Arizona), according to the manufacturer’s instructions. Primary antibody for p53 (clone DO-7, catalog No.M7001, DAKO, Denmark, Glostrup, 1:1000, Mouse monoclonal) was used. The p53 IHC staining were evaluated by two pathologists (JK and YNS) and classified into 3 categories: overexpression (strong diffuse nuclear immunoreactivity in all tumor cells), null (complete absence of nuclear immunoreactivity in all tumor cells), and usual (neither overexpression nor null, variable nuclear immunoreactivity in tumor cells).

### Statistical analysis

The R software (version 4.02 Vienna, Austria) was used to perform statistical analyses. Association between *TP53* mutational status by NGS test and IHC staining was tested using the χ2 and/or the Fisher exact tests. *P*-values less than 0.05 were considered statistically significant.

## Results

Malignant tumors of various organs were included in this study: anus in 2 (1.5%), brain in 7 (5.2%), breast in 6 (4.4%), large intestine in 107 (79.3%), lung in 2 (1.5%), ovary in 6 (4.4%), small intestine in 1 (0.7%), stomach in 2 (1.5%), and unknown in 2 (1.5%) cases (Table 1). Pathologic diagnosis of tumor was mostly adenocarcinoma (113 cases, 83.7%). Most cases were surgically resected specimens (125 cases, 92.6%) followed by forceps or needle biopsies (5 cases, 3.7%), and excisional biopsies (5 cases, 3.7%). Before obtaining tissue, neoadjuvant treatment was performed in 26 cases (19.3%).

**Table 1:** Clinicopathologic characteristics of cases

Characteristics	No of patients	% of patients
Sex		
Male	69	51.1
Female	66	48.9
Age		
< 60	55	40.7
≥ 60	80	59.3
Primary site		
Anus	2	1.5
Brain	7	5.2
Breast	6	4.4
Large intestine	107	79.3
Lung	2	1.5
Ovary	6	4.4
Small intestine	1	0.7
Stomach	2	1.5
Unknown	2	1.5
Diagnosis		
Adenocarcinoma	106	78.5
Carcinoma	2	1.5
Endometroid adenocarcinioma	1	0.7
Gastrointestinal stromal tumor	1	0.7
Glioblastoma	7	5.2
Invasive ductal carcinoma	6	4.4
Malignant brenner tumor	1	0.7
Mucinous aadenocarcinoma	6	4.4
Papillary serous carcinoma	4	3.0
Squamous cell carcinoma	1	0.7
Procedure		
Resection	125	92.6
Excision	5	3.7
Biopsy	5	3.7
Neoadjuvant treatment		
Yes	26	19.3
No	109	80.7

**Fig. 1:**
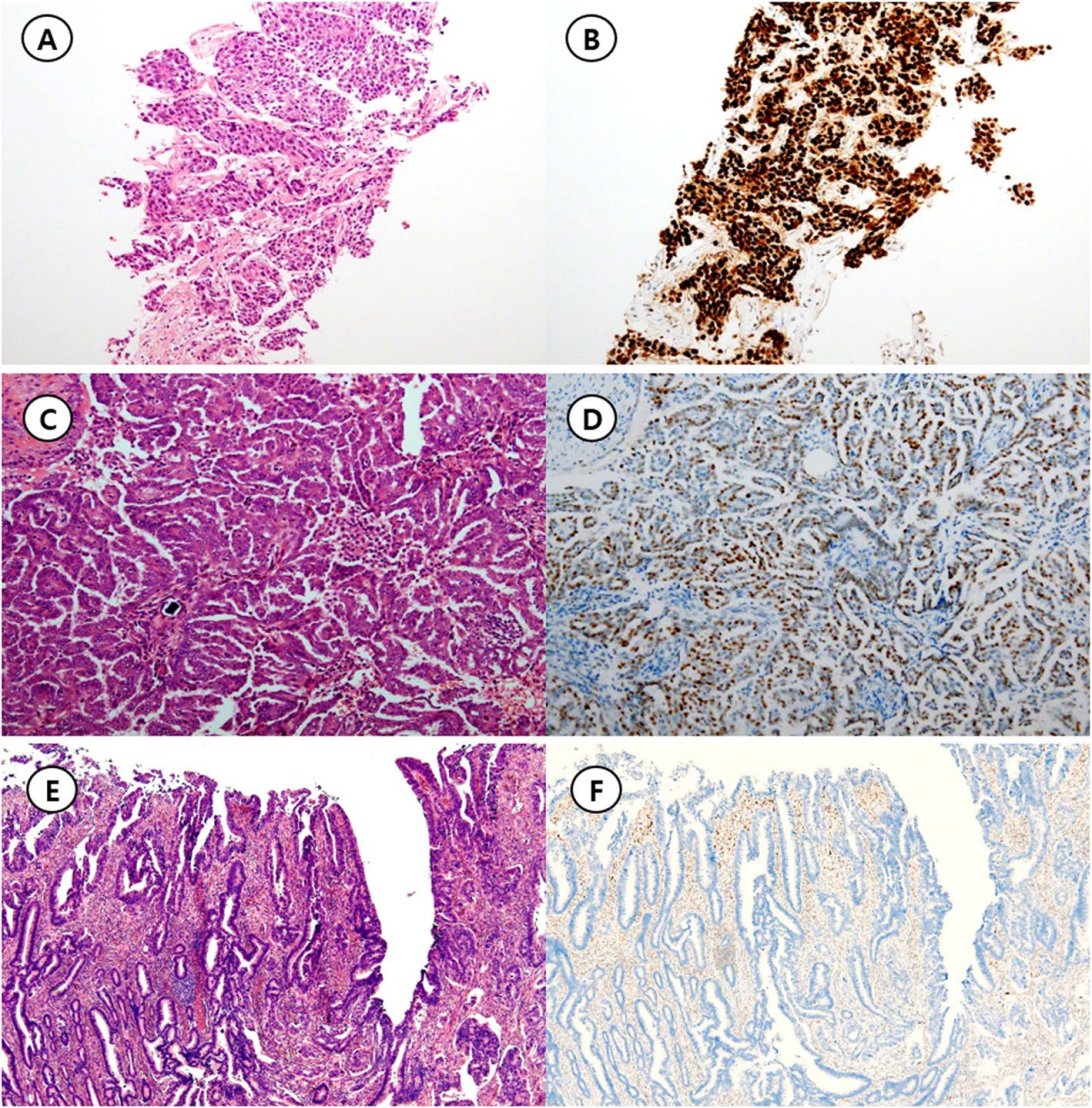
Representative figures for three p53 immunohistochemistry patterns. **A** Invasive ductal carcinoma of the breast harboring *TP53* H193R mutation (Hematoxylin & Eosin, × 1.25 objective lens). **B** Diffuse strong immunoreactivity for p53, that is overexpression pattern (p53 immunohistochemistry, × 1.25 objective lens). **C** Low grade papillary serous carcinoma of the ovary without any oncogenic *TP53* mutation (Hematoxylin & Eosin, × 10 objective lens) **D** Approximately a half of the tumor cells express p53 in the nuclei, that is usual pattern (p53 immunohistochemistry, × 10 objective lens). **E** Adenocarcinoma of the colon harboring *TP53* H214Qfs*2 mutation (Hematoxylin & Eosin, × 4 objective lens). **F** Tumor cells are completely negative for p53 protein expression. Adjacent non-neoplastic cells showing p53 expression serves as an internal positive control. This pattern is classified as null pattern (p53 immunohistochemistry, × 4 objective lens)

Through NGS analysis, *TP53* mutation was observed in 104 out of 135 cases (77.0%). Of the 104 cases with the *TP53* mutation, 72 types of *TP53* mutations were observed (Supplementary data). Table 2 shows the association of *TP53* mutational type and the IHC staining pattern. As a result of p53 IHC pattern, there were 86, 28 and 21 cases of overexpression, null, and usual pattern, respectively (Fig. 1). Most of the cases with p53 overexpression pattern except for 7 cases were accompanied by mutations (79/86, 91.9%) such as single nucleotide variation, In-frame indel, truncation, CN loss, and splice. Of the 7 cases without mutation, 4 cases received neo-adjuvant chemotherapy. Similarly, in the cases of p53 with null pattern, 25 out of 28 cases (89.3%) accompanied mutations. No mutations were observed in all cases (21/21, 100%) showing the p53 usual pattern.

**Table 2:** Immunohistochemical staining patterns and mutational analysis of *TP53*

**Mutation type**	**Immunohistochemical pattern of p53**			**Total**
	**Overexpression**	**Null type**	**Usual**	
**SNV**	65 (75.6)	0 (0)	0 (0)	65
**Frameshift indel**	0 (0)	7 (25)	0 (0)	7
**In-frame indel**	4 (4.7)	0 (0)	0 (0)	4
**Truncation**	1 (1.2)	9 (32.1)	0 (0)	10
**Copy-number loss**	1 (1.2)	0 (0)	0 (0)	1
**Splice**	2 (2.3)	7 (25)	0 (0)	9
**SNV/Frameshift indel**	1 (1.2)	1 (3.6)	0 (0)	2
**SNV/SNV**	5 (5.8)	0 (0)	0 (0)	5
**Splice/Truncation**	0 (0)	1 (3.6)	0 (0)	1
**Wild type**	7 (8.1)	3 (10.7)	21 (100)	31
**Total**	86	28	21	135

The corresponding percentages are shown in parentheses

The association of *TP53* mutation type and p53 IHC pattern was better observed when the *TP53* mutation were divided into IF-DBD and DIRUPTED according to the resulting sequence context (Table 3, *p* < 0.001). Because the in-frame SNVs or Indels are predicted to maintain most of the protein coding sequences intact while other alterations may disrupt amino acid sequences considerably, the p53 protein IHC pattern is predicted to be different between the two groups. Indeed, overexpression pattern was closely associated with the IF-DBD *TP53* mutations (sensitivity 100%, specificity 81.7%, positive predictive value 87.2%, negative predictive value 100%), while null pattern was associated with the DIRUPTED *TP53* mutations (sensitivity, 86.2%, specificity 97.2%, positive predictive value 89.3%, negative predictive value 96.3%). The usual p53 IHC pattern well predicted the absence of pathogenic *TP53* mutations (sensitivity 67.7%, specificity 100%, positive predictive value 100%, negative predictive value 91.2%). Additionally, among 26 cases that received previous neoadjuvant treatment, there were 19, 6, and 1 cases of overexpression, null type, and usual pattern, respectively. And the association between *TP53* mutation type and p53 IHC pattern was statistically significant (Table 4, *p* < 0.002).

**Table 3:** Immunohistochemical staining patterns and predicted functional consequence of *TP53 *mutations

Functional consequence	Immunohistochemical pattern of p53		
	**Overexpression**	**Null type**	**Usual**
**IF-DBD**	75 (87.2)	0 (0)	0 (0)
**DISRUPTED**	4 (4.7)	25 (89.3)	0 (0)
**Wild type**	7 (8.1)	3 (10.7)	21 (100)
**Total**	86	28	21

The corresponding percentages are shown in parentheses

**Table 4:** Immunohistochemical staining patterns and predicted functional consequence of *TP53* mutations within cases with previous neoadjuvant treatment

Functional consequence	Immunohistochemical pattern of p53		
	**Overexpression**	**Null type**	**Usual**
**IF-DBD**	14 (73.7)	0 (0)	0 (0)
**DISRUPTED**	1 (5.3)	4 (66.7)	0 (0)
**Wild type**	4 (2.1)	2 (33.3)	1 (100)
**Total**	19	6	1

The corresponding percentages are shown in parentheses

Table 5 shows the p53 IHC pattern according to the *MDM2* amplification status. The *MDM2*-amplified tumors showed more frequent usual p53 IHC pattern than *MDM2*-non-amplified tumors (*p* < 0.001, Fisher’s exact test). Null pattern was observed in only one case (1/12, 8.3%) in contrast to the assumption that p53 may be downregulated through *MDM2* amplification. Instead, when we looked at the presence and the type of *TP53* mutations in the *MDM2*-amplified tumors, the p53 IHC pattern just followed *TP53* mutation status regardless of *MDM2* amplification except for one case where p53 IHC overexpression pattern and wild type *TP53* mutation was observed (Table 6).

**Table 5:** Immunohisochemical staining pattern of p53 according to *MDM2* amplification status

	***MDM2* amplification status**	
	**No amplification**	**Amplification**
**Null type**	27 (22.0)	1 (8.3)^a^
**Usual**	13 (10.6)	8 (66.7)
**Overexpression**	83 (67.5)	3 (25.0)
**Total**	123	12

The corresponding percentages are shown in parentheses

^a^ This case harbored *TP53* compound R337C and E204Gfs*43 mutations and classified as DISRUPTED in main analysis

**Table 6:** Immunohistochemical staining patterns according to the *TP53* mutation types in *MDM2*-amplified cases

*TP53* mutation types	Immunohistochemical pattern of p53		
	**Overexpression**	**Null type**	**Usual**
**IF-DBD**	2 (66.7)	0 (0)	0 (0)
**DISDRUPTED**	0 (0)	1 (100)	0 (0)
**Wild type**	1 (33.3)	0 (0)	8 (100)
**Total**	3	1	8

The corresponding percentages are shown in parentheses

**Fig. 2:**
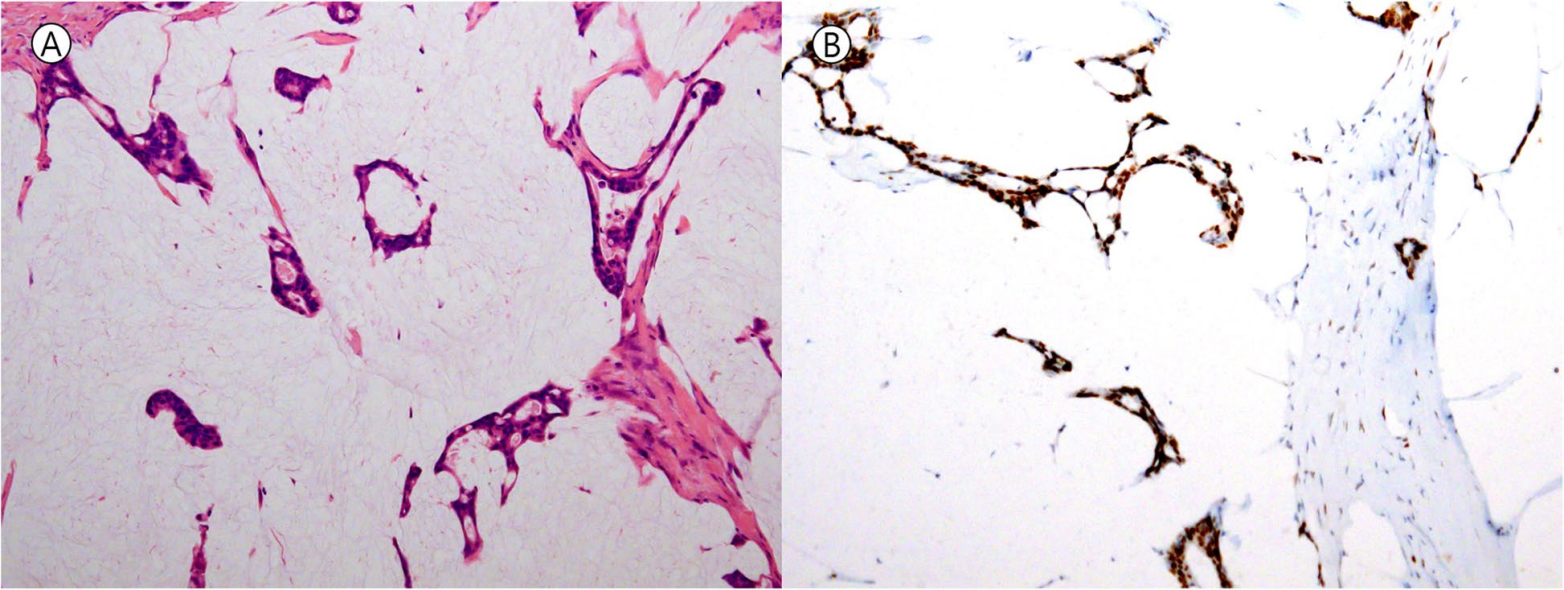
Mucinous adenocarcinoma of the colon that experienced neo-adjuvant chemoradiation therapy but did not show any *TP53* mutation. **A** Several strips or clusters of tumor cells are floating in the mucin pool (Hematoxylin & Eosin, × 10 objective lens). **B** TP53 overexpression pattern is noted (p53 immunohistochemistry, × 10 objective lens)

**Fig. 3:**
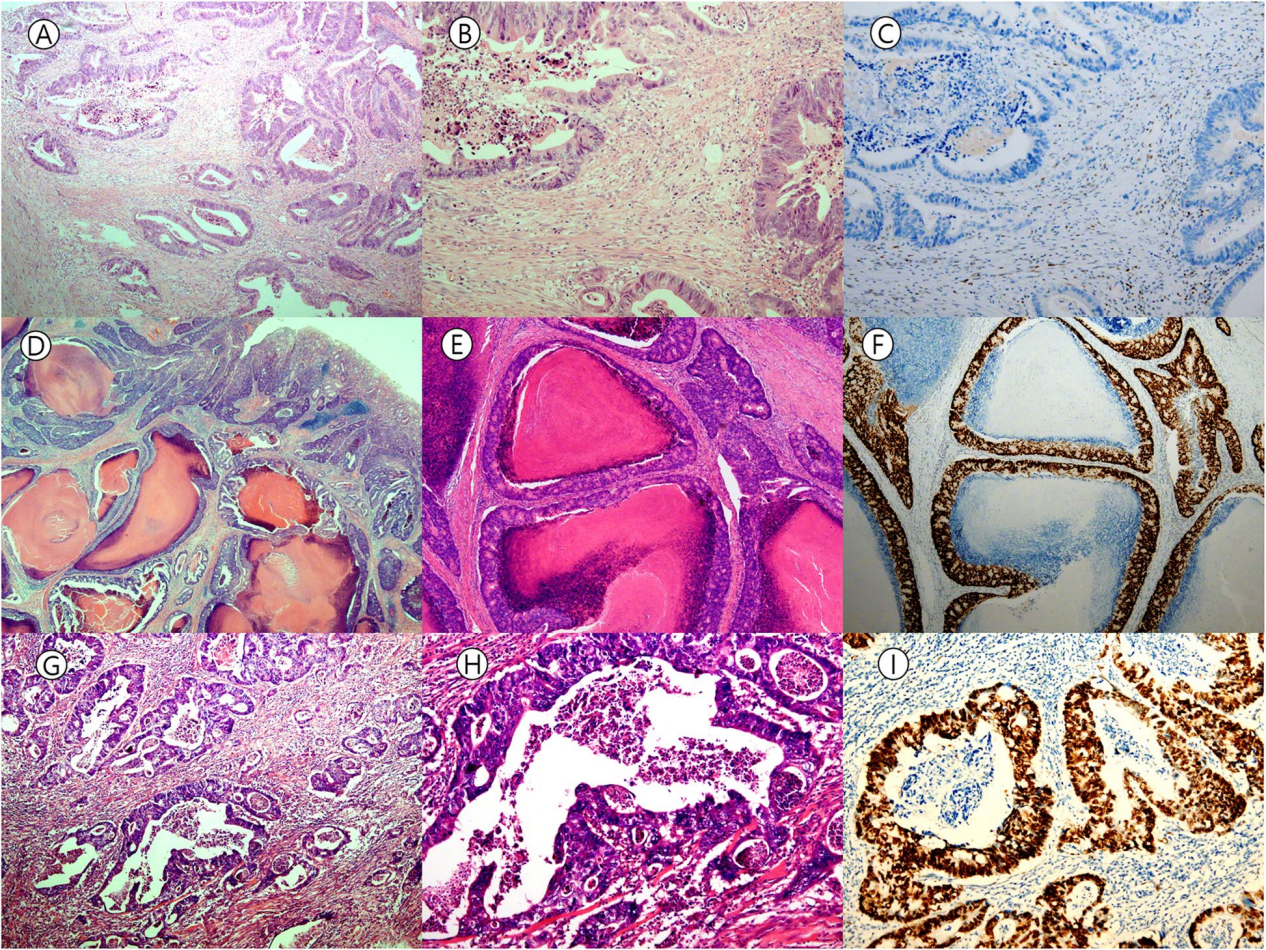
Representative case with discrepancy between *TP53* mutation and p53 IHC pattern. **A**&**B** Tumor area from which DNA was extracted for NGS analysis. Tumor cell purity is in acceptable range (Hematoxylin & Eosin, A: X4, B: X10 objective lens, respectively). **C** Null type pattern of p53 immunostaining is observed. No *TP53* mutation was detected in this case (p53 immunohistochemistry, × 10 objective lens). **D**, **E** Adenocarcinoma of the cecum with comedo-type necrosis. Area from which DNA has been extracted is shown in **E** (Hematoxylin & Eosin, D: X1.25, and E: X4 objective lens, respectively). **F** Overexpression pattern of p53 immunostaining is noted in the absence of detectable *TP53* mutation (p53 immunohistochemistry, × 4 objective lens). **G**, **H** Moderately differentiated adenocarcinoma of the rectum. DNA has been extracted from the area shown in **G** (Hematoxylin & Eosin, G: X4, and H: X10 objective lens, respectively). **I** Overexpression pattern of p53 immunostaining is noted in the absence of detectable *TP53* mutation. This patient did not receive neoadjuvant chemoradiation therapy (p53 immunohistochemistry, × 10 objective lens)

The p53 IHC pattern and *TP53* mutation discordance was observed in 14 out of 135 cases (14/135, 10.4%) (Table 7), and could be divided into the following 2 groups; 1) Wild type *TP53* gene showing either overexpression or null IHC pattern, and 2) DISRUPTED type *TP53* mutation showing overexpression pattern. In group 1, 7 cases showed overexpression and 3 cases showed null p53 IHC pattern in the absence of *TP53* mutations. Among the 7 cases showing p53 overexpression, 4 cases received neo-adjuvant chemoradiation therapy (Fig. 2). In addition, 2 out of 3 cases with p53 IHC null also received neo-adjuvant chemoradiation therapy. Since group 1 type discrepancies could also be seen due to failure of *TP53* mutation detection, we re-checked tumor cell purity. However, the tumor cell purity was within acceptable range in all cases (Fig. 3). The other cases belonged to the group 2: DISRUPTED type *TP53* mutation and overexpression p53 IHC pattern.

**Table 7:** p53 immunohistochemical staining pattern and *TP53* mutation discordant cases

	**Primary Site**	**Diagnosis**	**P53 IHC**	***TP53* mutation type**	**Class**	**alteration**	**allele frquency**	**cbioportal**	**MDM2 amplification**	**Neoadjuvant treatment**
**group 1**	Large intestine	Adenocarcinoma	NT	WT	WT	ND		negative	Not amplified	**Done**
	Large intestine	Adenocarcinoma	NT	WT	WT	ND		negative	Not amplified	**Done**
	Large intestine	Adenocarcinoma	NT	WT	WT	ND		negative	Not amplified	
	Large intestine	Adenocarcinoma	OE	WT	WT	ND		negative	Not amplified	
	Large intestine	Mucinous adenocarcinoma	OE	WT	WT	ND		negative	Not amplified	**Done**
	Large intestine	Adenocarcinoma	OE	WT	WT	ND		negative	Not amplified	
	Large intestine	Adenocarcinoma	OE	WT	WT	ND		negative	Not amplified	**Done**
	Anus	Adenocarcinoma	OE	WT	WT	ND		negative	Not amplified	**Done**
	Ovary	Papillary serous carcinoma	OE	WT	WT	ND		negative	Not amplified	**Done**
	Brain	Glioblastoma	OE	WT	WT	ND		negative	Amplified	
**group 2**	Large intestine	Adenocarcinoma	OE	truncation	LOF	R342*	0.23	Likely oncogenic	Not amplified	
	Large intestine	Adenocarcinoma	OE	splice site	LOF	X126_splice	0.33	Likely oncogenic	Not amplified	**Done**
	Large intestine	Adenocarcinoma	OE	CNV loss	LOF	loss	0	unknown	Not amplified	
	Large intestine	Adenocarcinoma	OE	splice site	LOF	X261_splice	0.31	Likely oncogenic	Not amplified	

*ND* Not detected, *NT* Null type, *OE* Overexpression, *WT* Wild type

## Discussion

In this study, the presence of oncogenic or likely oncogenic *TP53* mutations and their predicted functional consequences were well correlated with p53 IHC pattern in most cases. Sensitivity for p53 overexpression or null type pattern for prediction of IF-DBD type or DISRUPTED type *TP53* mutations was 100% and 86.2%, respectively, and specificity for TP53 usual pattern for prediction of WT *TP53* status was also high as 100%. *MDM2* amplification did not significantly affect TP53 IHC pattern, in contrast to the expectation that MDM2 would act as a negative regulator of p53, resulting in loss of p53 protein expression. Taken together, the p53 IHC pattern may serve as a reasonable surrogate for *TP53* mutations.

For the exceptions in those associations, some cases could be explained by predicted biological consequences. The 4 out of 7 cases with p53 overexpression in the absence of any *TP53* mutation received neo-adjuvant chemoradiation therapy. As physiologic p53 accumulation is possible due to the neo-adjuvant chemoradiation therapy, p53 overexpression may be observed in the absence of *TP53* gene mutations. However, the other occasions, such as p53 IHC null pattern in tumors harboring wild type *TP53*, p53 IHC overexpression pattern in tumors harboring DISRUPTED type *TP53* mutations, or p53 overexpression in chemoradiation-naïve tumors with wild type *TP53*, are hard to explain. There might be additional factors affecting p53 IHC pattern other than the presence of the type of *TP53* mutations that require further investigation.

As for the association between types of *TP53* mutations and p53 IHC patterns, IF-DBD *TP53* mutations were commonly associated with overexpression pattern while DISRUPTED *TP53* mutations were associated with null pattern. Those associations have been explained by the accumulated biological experiment data that p53 protein encoded by IF-DBD *TP53* mutations accumulate in the tumor cell nuclei through interfered MDM2-mediated ubiquitination, and DISRUPTED *TP53* mutations result in non-sense mediated decay, or prematurely truncated mRNA, and thus null pattern of p53 IHC [22]. Of note, *MDM2* amplification was not associated with p53 IHC null pattern, suggesting that *TP53* mutation itself may have a greater influence on IHC pattern than the role of MDM2 as a p53 negative regulator.

There have been several studies that attempted to correlate p53 IHC pattern with *TP53* mutational type [22, 23, 29, 30]. In the study of Yemelyanova et al. [23], the results of p53 IHC were divided into 5 groups according to the percentage of positive tumor cells, and when positive tumor cells were more than 60% or 0%, the sensitivity to detect *TP53* mutation was 94%. Additionally, in the study of Kobel et al. [22], the predictability of four types of p53 antibodies were evaluated by applying the same three-tier scoring system as in our study. Although it differed slightly depending on the type of antibody, the sensitivity and specificity for p53 IHC were 0.87 ~ 0.95 and 0.73 ~ 0.95 respectively. Singh et al. [29] also included cytoplasmic p53 IHC result as a mutant pattern, and the sensitivity and specificity for *TP53* mutation were 97.7% and 88.89% respectively. These studies, together with our study, showed that p53 IHC is useful for the prediction of *TP53* mutation type although not perfect.

Previous studies were mainly limited to one organ, but in this study, we could investigate p53 IHC patterns and *TP53* mutations in various organs, including colon, brain, breast, ovary, lung, stomach and small intestine, by virtue of NGS. In addition, we also reviewed discrepant cases and the reasons for the discrepancies. Although some discrepancies could not be reasonably explained, we found that neoadjuvant chemoradiation may induce p53 overexpression in the absence of IF-DBD type *TP53* mutations. Our study has some limitations. First, there may be selection bias due to availability of NGS data. In addition, the p53 IHC pattern was evaluated by only one type of monoclonal antibody, and detailed mechanistic study for discrepancies between p53 IHC pattern and *TP53* mutation type could not be performed because of shortage of time and resources.

In conclusion, *TP53* mutational status can be well predicted through three p53 IHC patterns (overexpression, null, and usual) in most cases: 1) overexpression for IF-DBD type *TP53* mutations, 2) null for DISRUPTED type *TP53* mutations, and 3) usual for the absence of *TP53* mutations. In addition, *MDM2* amplification does not seem to significantly affect the p53 IHC pattern. Finally, we propose that pattern-based approach (overexpression, null, and usual patterns) might be more informative in the interpretation of p53 IHC than traditional interpretation system based on positive tumor cell proportion.

## Supplementary Information

**Additional file 1: Supplementary data. **Detailed p53 mutational features in all 135 cases.

## Abbreviations

IHC: Immunohistochemical staining
DISRUPTED: Truncations, frameshifts, splice site mutations, and deep deletions
IF-DBD: In-frame mutations in the DNA binding domain
NGS: Next-generation sequencing
