# Supplementary material for: p53 immunostaining pattern is a useful surrogate marker for TP53 gene mutations
Source: Diagn Pathol. 2022 Dec 5;17:92. doi: 10.1186/s13000-022-01273-w (PMC9720942; doi:10.1186/s13000-022-01273-w)
Supplement: Supplementary file 1 — Additional file 1: Supplementary data. Detailed p53 mutational features in all 135 cases. [file 13000_2022_1273_MOESM1_ESM.docx]

**Supplementary data.** Detailed p53 mutational features in all 135 cases

| Case # | Primary Site | Diagnosis | P53 IHC | p53 type | Alteration | TP53 class | Allele frequency | Depth of coverage***** | MDM2 amplification | Neoadjuvant Tx |
| --- | --- | --- | --- | --- | --- | --- | --- | --- | --- | --- |
| 1 | Large intestine | Adenocarcinoma | Null type | Frameshift indel | V73Rfs*76 | DISRUPTED | 0.3 | 120x | No amplification | Yes |
| 2 | Large intestine | Adenocarcinoma | Null type | Wild type | Wild type | Wild type |  | 160x | No amplification | Yes |
| 3 | Large intestine | Adenocarcinoma | Null type | Truncation | R213* | DISRUPTED | 0.31 | 158x | No amplification | No |
| 4 | Large intestine | Adenocarcinoma | Null type | Splice | X255_splice | DISRUPTED | 0.31 | 118x | No amplification | No |
| 5 | Large intestine | Adenocarcinoma | Null type | Splice | X187_splice | DISRUPTED | 0.16 | 171x | No amplification | Yes |
| 6 | Large intestine | Adenocarcinoma | Null type | Wild type | Wild type | Wild type |  | 303x | No amplification | Yes |
| 7 | Large intestine | Adenocarcinoma | Null type | Truncation | R196* | DISRUPTED | 0.3 | 109x | No amplification | Yes |
| 8 | Large intestine | Mucinous adenocarcinoma | Null type | Truncation | S121* | DISRUPTED | 0.47 | 40x | No amplification | No |
| 9 | Large intestine | Adenocarcinoma | Null type | Frameshift indel | W91Gfs*32 | DISRUPTED | 0.12 | 129x | No amplification | Yes |
| 10 | Large intestine | Adenocarcinoma | Null type | Frameshift indel | H214Qfs*7 | DISRUPTED | 0.45 | 146x | No amplification | No |
| 11 | Large intestine | Adenocarcinoma | Null type | Frameshift indel | K320Efs*17 | DISRUPTED | 0.1 | 73x | No amplification | No |
| 12 | Large intestine | Adenocarcinoma | Null type | Wild type | Wild type | Wild type |  | 238x | No amplification | No |
| 13 | Large intestine | Adenocarcinoma | Null type | Frameshift indel | H214Qfs*2 | DISRUPTED | 0.31 | 329x | No amplification | No |
| 14 | Large intestine | Adenocarcinoma | Null type | Frameshift indel | R110Vfs*13 | DISRUPTED | 0.33 | 76x | No amplification | No |
| 15 | Large intestine | Adenocarcinoma | Null type | Truncation | R213* | DISRUPTED | 0.47 | 190x | No amplification | No |
| 16 | Large intestine | Adenocarcinoma | Null type | Splice | X331_splice | DISRUPTED | 0.5 | 229x | No amplification | No |
| 17 | Large intestine | Adenocarcinoma | Null type | Frameshift indel | C124Afs*46 | DISRUPTED | 0.66 | 61x | No amplification | No |
| 18 | Unknown | Carcinoma | Null type | Splice | X225_splice | DISRUPTED | 0.67 | 200x | No amplification | No |
| 19 | Large intestine | Adenocarcinoma | Null type | Truncation | R213* | DISRUPTED | 0.14 | 391x | No amplification | No |
| 20 | Large intestine | Adenocarcinoma | Null type | Truncation/Splice | R213*/X125_splice | DISRUPTED | 0.3/0.27 | 351x/75x | No amplification | No |
| 21 | Large intestine | Adenocarcinoma | Null type | Splice | X255_splice | DISRUPTED | 0.41 | 103x | No amplification | No |
| 22 | Large intestine | Adenocarcinoma | Null type | Truncation | S183* | DISRUPTED | 0.47 | 253x | No amplification | No |
| 23 | Large intestine | Adenocarcinoma | Null type | Splice | X331_splice | DISRUPTED | 0.46 | 165x | No amplification | No |
| 24 | Large intestine | Adenocarcinoma | Null type | Truncation | W146* | DISRUPTED | 0.45 | 144x | No amplification | No |
| 25 | Large intestine | Adenocarcinoma | Null type | Truncation | R213* | DISRUPTED | 0.34 | 241x | No amplification | No |
| 26 | Large intestine | Mucinous adenocarcinoma | Null type | Truncation | R213* | DISRUPTED | 0.38 | 278x | No amplification | No |
| 27 | Breast | Invasive ductal carcinoma | Null type | Splice | X125_splice | DISRUPTED | 0.55 | 58x | No amplification | No |
| 28 | Large intestine | Adenocarcinoma | Usual | Wild type | Wild type | Wild type |  | 201x | No amplification | No |
| 29 | Large intestine | Adenocarcinoma | Usual | Wild type | Wild type | Wild type |  | 134x | No amplification | Yes |
| 30 | Large intestine | Adenocarcinoma | Usual | Wild type | Wild type | Wild type |  | 185x | No amplification | No |
| 31 | Large intestine | Adenocarcinoma | Overexpression | Truncation | R342* | DISRUPTED | 0.23 | 127x | No amplification | No |
| 32 | Large intestine | Adenocarcinoma | Usual | Wild type | Wild type | Wild type |  | 259x | No amplification | No |
| 33 | Unknown | Carcinoma | Usual | Wild type | Wild type | Wild type |  | 295x | No amplification | No |
| 34 | Large intestine | Adenocarcinoma | Usual | Wild type | Wild type | Wild type |  | 104x | No amplification | No |
| 35 | Large intestine | Adenocarcinoma | Usual | Wild type | Wild type | Wild type |  | 336x | No amplification | No |
| 36 | Large intestine | Adenocarcinoma | Usual | Wild type | Wild type | Wild type |  | 256x | No amplification | No |
| 37 | Ovary | Papillary serous carcinoma | Usual | Wild type | Wild type | Wild type |  | 233x | No amplification | No |
| 38 | Large intestine | Adenocarcinoma | Usual | Wild type | Wild type | Wild type |  | 221x | No amplification | No |
| 39 | Large intestine | Adenocarcinoma | Usual | Wild type | Wild type | Wild type |  | 200x | No amplification | No |
| 40 | Small intestine | Gastrointestinal stromal tumor | Usual | Wild type | Wild type | Wild type |  | 220x | No amplification | No |
| 41 | Large intestine | Adenocarcinoma | Overexpression | SNV | A161T | IF-DBD | 0.39 | 102x | No amplification | No |
| 42 | Large intestine | Mucinous adenocarcinoma | Overexpression | SNV | R248W | IF-DBD | 0.35 | 126x | No amplification | Yes |
| 43 | Large intestine | Adenocarcinoma | Overexpression | Splice | X126_splice | DISRUPTED | 0.33 | 103x | No amplification | Yes |
| 44 | Large intestine | Adenocarcinoma | Overexpression | Wild type | Wild type | Wild type |  | 66x | No amplification | No |
| 45 | Anus | Mucinous adenocarcinoma | Overexpression | SNV | R175H | IF-DBD | 0.06 | 131x | No amplification | Yes |
| 46 | Large intestine | Adenocarcinoma | Overexpression | SNV | C176R | IF-DBD | 0.28 | 99x | No amplification | No |
| 47 | Large intestine | Adenocarcinoma | Overexpression | SNV | R249S | IF-DBD | 0.22 | 139x | No amplification | Yes |
| 48 | Ovary | Papillary serous carcinoma | Overexpression | SNV | Y236C | IF-DBD | 0.6 | 112x | No amplification | No |
| 49 | Large intestine | Adenocarcinoma | Overexpression | SNV | R248Q | IF-DBD | 0.07 | 154x | No amplification | Yes |
| 50 | Large intestine | Adenocarcinoma | Overexpression | SNV | R282W | IF-DBD | 0.46 | 137x | No amplification | No |
| 51 | Large intestine | Adenocarcinoma | Overexpression | SNV | R273C | IF-DBD | 0.16 | 132x | No amplification | Yes |
| 52 | Large intestine | Adenocarcinoma | Overexpression | SNV | L252P | IF-DBD | 0.43 | 249x | No amplification | No |
| 53 | Large intestine | Adenocarcinoma | Overexpression | SNV | R248W | IF-DBD | 0.4 | 142x | No amplification | No |
| 54 | Large intestine | Adenocarcinoma | Overexpression | SNV | P278A | IF-DBD | 0.09 | 282x | No amplification | Yes |
| 55 | Large intestine | Adenocarcinoma | Overexpression | SNV/SNV | R273H/V173M | IF-DBD | 0.18/0.10 | 222x/190x | No amplification | Yes |
| 56 | Large intestine | Adenocarcinoma | Overexpression | SNV | R175H | IF-DBD | 0.46 | 149x | No amplification | No |
| 57 | Large intestine | Adenocarcinoma | Overexpression | SNV | Y163C | IF-DBD | 0.27 | 111x | No amplification | Yes |
| 58 | Large intestine | Adenocarcinoma | Overexpression | SNV | R248W | IF-DBD | 0.2 | 92x | No amplification | No |
| 59 | Anus | Adenocarcinoma | Overexpression | Wild type | Wild type | Wild type |  | 334x | No amplification | Yes |
| 60 | Large intestine | Adenocarcinoma | Overexpression | SNV | G245S | IF-DBD | 0.5 | 122x | No amplification | No |
| 61 | Large intestine | Adenocarcinoma | Overexpression | SNV | I255S | IF-DBD | 0.12 | 193x | No amplification | No |
| 62 | Ovary | Papillary serous carcinoma | Overexpression | SNV | I195T | IF-DBD | 0.46 | 163x | No amplification | No |
| 63 | Large intestine | Adenocarcinoma | Overexpression | Copy-number loss | loss | DISRUPTED | 0 | 228x | No amplification | No |
| 64 | Large intestine | Adenocarcinoma | Overexpression | SNV | Y236D | IF-DBD | 0.32 | 75x | No amplification | No |
| 65 | Large intestine | Adenocarcinoma | Overexpression | Splice | X261_splice | DISRUPTED | 0.31 | 137x | No amplification | No |
| 66 | Large intestine | Adenocarcinoma | Overexpression | SNV | H179Q | IF-DBD | 0.44 | 196x | No amplification | No |
| 67 | Large intestine | Adenocarcinoma | Overexpression | In-frame indel | P250_I251del | IF-DBD | 0.07 | 108x | No amplification | No |
| 68 | Large intestine | Adenocarcinoma | Overexpression | SNV | G244S | IF-DBD | 0.21 | 208x | No amplification | No |
| 69 | Large intestine | Mucinous adenocarcinoma | Overexpression | Wild type | Wild type | Wild type |  | 258x | No amplification | Yes |
| 70 | Breast | Invasive ductal carcinoma | Overexpression | SNV | H193R | IF-DBD | 0.73 | 135x | No amplification | No |
| 71 | Large intestine | Adenocarcinoma | Overexpression | SNV | R175H | IF-DBD | 0.3 | 322x | No amplification | Yes |
| 72 | Large intestine | Adenocarcinoma | Overexpression | SNV | S127F | IF-DBD | 0.48 | 97x | No amplification | No |
| 73 | Large intestine | Adenocarcinoma | Overexpression | SNV | C242G | IF-DBD | 0.44 | 101x | No amplification | No |
| 74 | Large intestine | Adenocarcinoma | Overexpression | SNV/SNV | G245D/C176Y | IF-DBD | 0.24/0.51 | 202x/219x | No amplification | No |
| 75 | Large intestine | Adenocarcinoma | Overexpression | SNV/SNV | Q331H/R282W | IF-DBD | 0.18 | 146x | No amplification | No |
| 76 | Large intestine | Adenocarcinoma | Overexpression | SNV | R175H | IF-DBD | 0.09 | 89x | No amplification | No |
| 77 | Large intestine | Adenocarcinoma | Overexpression | SNV | Y236C | IF-DBD | 0.49 | 83x | No amplification | No |
| 78 | Large intestine | Adenocarcinoma | Overexpression | In-frame indel | P191del | IF-DBD | 0.3 | 70x | No amplification | No |
| 79 | Large intestine | Adenocarcinoma | Overexpression | SNV | I162S | IF-DBD | 0.25 | 166x | No amplification | No |
| 80 | Large intestine | Adenocarcinoma | Overexpression | SNV/Frameshift indel | R248Q/C242Afs*5 | IF-DBD | 0.1/0.32 | 163x/165x | No amplification | No |
| 81 | Large intestine | Adenocarcinoma | Overexpression | SNV | C176G | IF-DBD | 0.84 | 117x | No amplification | Yes |
| 82 | Large intestine | Adenocarcinoma | Overexpression | SNV | R282W | IF-DBD | 0.4 | 289x | No amplification | No |
| 83 | Breast | Invasive ductal carcinoma | Overexpression | SNV | M237I | IF-DBD | 0.7 | 105x | No amplification | No |
| 84 | Large intestine | Adenocarcinoma | Overexpression | SNV | R273H | IF-DBD | 0.2 | 230x | No amplification | No |
| 85 | Large intestine | Adenocarcinoma | Overexpression | SNV | G245V | IF-DBD | 0.3 | 180x | No amplification | No |
| 86 | Large intestine | Adenocarcinoma | Overexpression | SNV | R175H | IF-DBD | 0.54 | 126x | No amplification | No |
| 87 | Large intestine | Adenocarcinoma | Overexpression | SNV | R273C | IF-DBD | 0.51 | 273x | No amplification | No |
| 88 | Large intestine | Adenocarcinoma | Overexpression | SNV | R248Q | IF-DBD | 0.47 | 188x | No amplification | No |
| 89 | Large intestine | Adenocarcinoma | Overexpression | SNV | R273C | IF-DBD | 0.56 | 187x | No amplification | No |
| 90 | Large intestine | Adenocarcinoma | Overexpression | SNV | R175H | IF-DBD | 0.48 | 157x | No amplification | No |
| 91 | Large intestine | Adenocarcinoma | Overexpression | SNV | R273H | IF-DBD | 0.5 | 173x | No amplification | No |
| 92 | Large intestine | Adenocarcinoma | Overexpression | SNV | R282W | IF-DBD | 0.39 | 130x | No amplification | No |
| 93 | Large intestine | Adenocarcinoma | Overexpression | SNV | R282W | IF-DBD | 0.25 | 255x | No amplification | No |
| 94 | Ovary | Endometroid adenocarcinoma | Overexpression | SNV | G245V | IF-DBD | 0.73 | 193x | No amplification | No |
| 95 | Large intestine | Adenocarcinoma | Overexpression | Wild type | Wild type | Wild type |  | 243x | No amplification | No |
| 96 | Large intestine | Adenocarcinoma | Overexpression | SNV | R273C | IF-DBD | 0.52 | 190x | No amplification | No |
| 97 | Large intestine | Adenocarcinoma | Overexpression | SNV | R273H | IF-DBD | 0.68 | 227x | No amplification | No |
| 98 | Large intestine | Adenocarcinoma | Overexpression | SNV | L257P | IF-DBD | 0.34 | 106x | No amplification | No |
| 99 | Large intestine | Adenocarcinoma | Overexpression | SNV | E258G | IF-DBD | 0.4 | 195x | No amplification | No |
| 100 | Large intestine | Adenocarcinoma | Overexpression | SNV | P151T | IF-DBD | 0.22 | 221x | No amplification | No |
| 101 | Large intestine | Adenocarcinoma | Overexpression | SNV | Y220C | IF-DBD | 0.33 | 309x | No amplification | No |
| 102 | Large intestine | Adenocarcinoma | Overexpression | SNV/SNV | R273H/G199R | IF-DBD | 0.22/0.21 | 209x/218x | No amplification | No |
| 103 | Large intestine | Adenocarcinoma | Overexpression | SNV | G245S | IF-DBD | 0.5 | 141x | No amplification | No |
| 104 | Large intestine | Adenocarcinoma | Overexpression | SNV | R273C | IF-DBD | 0.33 | 310x | No amplification | No |
| 105 | Large intestine | Adenocarcinoma | Overexpression | SNV | G199V | IF-DBD | 0.47 | 200x | No amplification | No |
| 106 | Large intestine | Adenocarcinoma | Overexpression | SNV | R175H | IF-DBD | 0.44 | 212x | No amplification | No |
| 107 | Large intestine | Adenocarcinoma | Overexpression | SNV | G266R | IF-DBD | 0.37 | 163x | No amplification | No |
| 108 | Large intestine | Adenocarcinoma | Overexpression | SNV | H214R | IF-DBD | 0.62 | 190x | No amplification | No |
| 109 | Large intestine | Adenocarcinoma | Overexpression | SNV | V274A | IF-DBD | 0.3 | 272x | No amplification | No |
| 110 | Large intestine | Adenocarcinoma | Overexpression | SNV | E286G | IF-DBD | 0.33 | 430x | No amplification | No |
| 111 | Large intestine | Adenocarcinoma | Overexpression | In-frame indel | P177_C182dup | IF-DBD | 0.15 | 267x | No amplification | No |
| 112 | Large intestine | Adenocarcinoma | Overexpression | SNV | F113S | IF-DBD | 0.13 | 52x | No amplification | No |
| 113 | Large intestine | Adenocarcinoma | Overexpression | SNV | [R282W](mailto:R282@) | IF-DBD | 0.28 | 145x | No amplification | No |
| 114 | Large intestine | Adenocarcinoma | Overexpression | SNV/SNV | G244D/Y126D | IF-DBD | 0.08/0.24 | 245x/233x | No amplification | No |
| 115 | Large intestine | Adenocarcinoma | Overexpression | SNV | R273C | IF-DBD | 0.2 | 137x | No amplification | No |
| 116 | Ovary | Papillary serous carcinoma | Overexpression | Wild type | Wild type | Wild type |  | 233x | No amplification | Yes |
| 117 | Large intestine | Adenocarcinoma | Overexpression | SNV | R273H | IF-DBD | 0.6 | 157x | No amplification | No |
| 118 | Large intestine | Adenocarcinoma | Overexpression | SNV | R175H | IF-DBD | 0.37 | 249x | No amplification | No |
| 119 | Breast | Invasive ductal carcinoma | Overexpression | SNV | R175H | IF-DBD | 0.5 | 151x | No amplification | Yes |
| 120 | Breast | Invasive ductal carcinoma | Overexpression | SNV | R282W | IF-DBD | 0.48 | 329x | No amplification | Yes |
| 121 | Breast | Invasive ductal carcinoma | Overexpression | In-frame indel | I255del | IF-DBD | 0.27 | 156x | No amplification | Yes |
| 122 | Large intestine | Adenocarcinoma | Overexpression | Wild type | Wild type | Wild type |  | 246x | No amplification | Yes |
| 123 | Large intestine | Mucinous adenocarcinoma | Usual | Wild type | Wild type | Wild type |  | 254x | No amplification | No |
| 124 | Lung | Adenocarcinoma | Usual | Wild type | Wild type | Wild type |  | 224x | Amplification | No |
| 125 | Stomach | Adenocarcinoma | Usual | Wild type | Wild type | Wild type |  | 481x | Amplification | No |
| 126 | Brain | Glioblastoma | Usual | Wild type | Wild type | Wild type |  | 256x | Amplification | No |
| 127 | Brain | Glioblastoma | Overexpression | Wild type | Wild type | Wild type |  | 313x | Amplification | No |
| 128 | Brain | Glioblastoma | Usual | Wild type | Wild type | Wild type |  | 297x | Amplification | No |
| 129 | Lung | Squamous cell carcinoma | Overexpression | SNV | E285K | IF-DBD | 0.51 | 199x | Amplification | No |
| 130 | Brain | Glioblastoma | Usual | Wild type | Wild type | Wild type |  | 489x | Amplification | No |
| 131 | Brain | Glioblastoma | Usual | Wild type | Wild type | Wild type |  | 392x | Amplification | No |
| 132 | Stomach | Adenocarcinoma | Overexpression | SNV | A276G | IF-DBD | 0.56 | 544x | Amplification | Yes |
| 133 | Brain | Glioblastoma | Null type | SNV/Frameshift indel | R337C/E204Gfs*43 | DISRUPTED | 0.04/0.11 | 289x | Amplification | No |
| 134 | Ovary | Malignant Brenner tumor | Usual | Wild type | Wild type | Wild type |  | 407x | Amplification | No |
| 135 | Brain | Glioblastoma | Usual | Wild type | Wild type | Wild type |  | 430x | Amplification | No |

* Depth of coverage is expressed as local depth of coverage for *TP53* mutant cases and mean target coverage for *TP53* wild type cases. All depth of coverage data is post-de-duplication value.

**<Figure legends>**

**Figure 1 Representative figures for three p53 immunohistochemistry patterns.** (A) Invasive ductal carcinoma of the breast harboring *TP53* H193R mutation (Hematoxylin & Eosin, x1.25 objective lens). (B) Diffuse strong immunoreactivity for p53, that is overexpression pattern (p53 immunohistochemistry, x1.25 objective lens). (C) Low grade papillary serous carcinoma of the ovary without any oncogenic *TP53* mutation (Hematoxylin & Eosin, x10 objective lens) (D) Approximately a half of the tumor cells express p53 in the nuclei, that is usual pattern (p53 immunohistochemistry, x10 objective lens). (E) Adenocarcinoma of the colon harboring *TP53* H214Qfs*2 mutation (Hematoxylin & Eosin, x4 objective lens). (F) Tumor cells are completely negative for p53 protein expression. Adjacent non-neoplastic cells showing p53 expression serves as an internal positive control. This pattern is classified as null pattern (p53 immunohistochemistry, x4 objective lens).

**Figure 2 Mucinous adenocarcinoma of colon that experienced neo-adjuvant chemoradiation therapy but did not show any *TP53* mutation.** (A) Several strips or clusters of tumor cells are floating in the mucin pool (Hematoxylin & Eosin, x10 objective lens). (B) TP53 overexpression pattern is noted (p53 immunohistochemistry, x10 objective lens).

**Figure 3 Representative case with discrepancy between *TP53* mutation and p53 IHC pattern.** (A&B) Tumor area from which DNA was extracted for NGS analysis. Tumor cell purity is in acceptable range (Hematoxylin & Eosin, A: X4, B: X10 objective lens, respectively). (C) Null type pattern of p53 immunostaining is observed. No *TP53* mutation was detected in this case (p53 immunohistochemistry, x10 objective lens). (D, E) Adenocarcinoma of the cecum with comodo-type necrosis. Area from which DNA has been extracted is shown in (E) (Hematoxylin & Eosin, D: X1.25, and E: X4 objective lens, respectively). (F) Overexpression pattern of p53 immunostaining is noted in the absence of detectable *TP53* mutation (p53 immunohistochemistry, x4 objective lens). (G, H) Moderately differentiated adenocarcinoma of the rectum. DNA has been extracted from the area shown in (G) (Hematoxylin & Eosin, G: X4, and H: X10 objective lens, respectively). (I) Overexpression pattern of p53 immunostaining is noted in the absence of detectable *TP53* mutation. This patient did not receive neoadjuvant chemoradiation therapy (p53 immunohistochemistry, x10 objective lens).
